# Detection of Adhesion Molecules on Inflamed Macrophages at Early-Stage Using SERS Probe Gold Nanorods

**DOI:** 10.1007/s40820-016-0111-7

**Published:** 2016-09-23

**Authors:** Dakrong Pissuwan, Yusuke Hattori

**Affiliations:** 1 World Premier International Immunology Frontier Research Center, Osaka University, Osaka, 5650871 Japan; 2 Materials Science and Engineering Program, Multidisciplinary Unit, Faculty of Science, Mahidol University, Bangkok, 10400 Thailand; 3 Research Institute of Pharmaceutical Sciences, Musashino University, Tokyo, 2028585 Japan

**Keywords:** Gold nanorod, SERS, Inflamed cell, Macrophage, ICAM-1

## Abstract

In recent years, it has been shown that inflammatory biomarkers can be used as an effective signal for disease diagnoses. The early detection of these signals provides useful information that could prevent the occurrence of severe diseases. Here, we employed surface-enhanced Raman scattering (SERS) probe gold nanorods (GNRs) as a tool for the early detection of inflammatory molecules in inflamed cells. A murine macrophage cell line (Raw264.7) stimulated with lipopolysaccharide (LPS) was used as a model in this study. The prepared SERS probe GNRs containing 4-mercaptobenzoic acid as a Raman reporter to generate SERS signals were used for detection of intracellular adhesion molecule-1 (ICAM-1) in macrophages after treatment with LPS for varying lengths of time. Our results show that SERS probe GNRs could detect significant differences in the expression of ICAM-1 molecules in LPS-treated macrophages compared to those in untreated macrophages after only 1 h of LPS treatment. In contrast, when using fluorescent labeling or enzyme-linked immunosorbent assays (ELISA) to detect ICAM-1, significant differences between inflamed and un-inflamed macrophages were not seen until the cells had been treated with LPS for 5 h. These results indicate that our SERS probe GNRs provide a higher sensitivity for detecting biomarker molecules in inflamed macrophages than the conventional fluorescence and ELISA techniques, and could therefore be useful as a potential diagnostic tool for managing disease risk.

**Electronic supplementary material:**

The online version of this article (doi:10.1007/s40820-016-0111-7) contains supplementary material, which is available to authorized users.

## Introduction

Excessive inflammation can contribute to diseases such as cancer, asthma, rheumatoid arthritis, Alzheimer’s disease, and atherosclerosis. Inflammation has been reported as a vital factor in causing atherosclerosis in particular [1], induced by inflammatory cytokines such as interleukin-1 (IL-1), tumor necrosis factor α (TNF-α), and interferon-γ (IFN-γ) [2–4]. These cytokines stimulate the expression of adhesion molecules such as intercellular adhesion molecule-1 (ICAM-1) in macrophages and in endothelial cells. The expression of ICAM-1 in these cells could lead to many diseases. Therefore, to prevent severe diseases, it is important to detect the early events of diseases. ICAM-1 expression could be used as an effective indicator for clinical diagnosis.

Various approaches have been used to detect cellular inflammation or inflammatory response molecules such as molecular imaging with fluorescent dyes [5], enzyme-linked immunosorbent assay (ELISA) [6], and ultrasound [7]. However, these techniques have not been sensitive enough to detect the biomolecular changes during atherogenesis at an early stage [8, 9]. Consequently, there has been a continued effort to develop a way of detecting this change in inflamed cells or in biological tissues. Recently, gold nanorods (GNRs) have shown a high potential for use in various biological applications because of their optical properties and friendly surface chemistry, which allows for attachment with several types of molecules [10–14]. It is well known that GNRs have two plasmon bands: transverse and longitudinal. With longitudinal plasmon resonances, GNRs provide a higher light absorption and scattering than plasmon resonances from other shapes of gold nanoparticles, such as spherical shapes or nanoshells [15, 16]. This property makes GNRs potentially suitable for enhancing Raman signals in a phenomenon known as surface-enhanced Raman scattering (SERS). It was reported that the combination of SERS with GNRs can increase sensitivity and efficiency for detection of targeted molecules [17]. Furthermore, it was found that GNRs provide a higher sensitivity for SERS detection than nanospheres due to the properties discussed above [13, 18]. Therefore, the use of GNRs as SERS agents for detection of inflamed cells could be an effective approach for medical diagnosis of inflammatory diseases.

Several groups have used GNRs with Raman reporters to promote SERS techniques for immunological and biological assays [18–22]. Although there have been some reports of using SERS nanoparticle techniques for detection of inflammatory biomarkers, including one report specifically related to atherosclerosis [23], to our knowledge, previous studies have only demonstrated how strong Raman signals could be detected using their designed SERS particles. However, there is no investigation of the time stage of diagnosis. Furthermore, former studies examined the detection of signals from inflamed endothelial cells. However, it was reported that the expression of ICAM-1 in LPS stimulated macrophages involves the induction of a plaque-like formation, which later leads to cell aggregation [24]. The increase of ICAM-1 expression also promotes the interaction of macrophages with the endothelium, which eventually leads to inflammation [25]. Therefore, the detection of ICAM-1 expression in macrophage cells could help predict the development of cell inflammation.

Several reports have demonstrated that the combination of GNRs with Raman molecules can be used as a SERS substrate for immunoassays [20, 26] or used for targeting specific biomarkers [27]. Wu et al. [20] synthesized gold/silver core–shell nanorods (aspect ratio ~3.0), incorporated them with a reporter molecule [4-mercaptobenzoic acid (4MBA)] as a SERS probe, and prepared the probe as a substrate on a glass slide for use in an immunoassay. Their results showed that the prepared gold/silver core–shell nanorod probe on the substrate could detect human immunoglobulin G (IgG) in ranges from 700 nM to 70 fM.

**Fig. 1:**
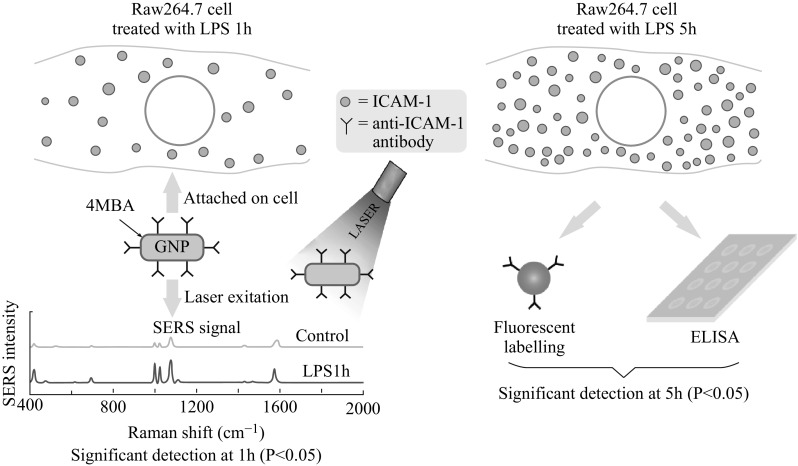
Schematic drawing representing the use of SERS probe GNR techniques compared with ELISA and fluorescent labeling techniques for detection of adhesion molecules expressed on the surface of macrophage cells (Raw264.7)

Using SERS to detect immunological changes directly on cells is very rare. Most published studies prepared the SERS substrate and then used it as a sensor for detecting molecules outside of cells. Here, we demonstrate the approach of using GNR-based SERS to directly detect the expression of ICAM-1 molecules on inflamed macrophages at different stages of stimulation by modifying the approach described by Wu et al. [20]. We used RAW264.7 cells with and without treatment with LPS as a model cell in this study. To our knowledge, there have been no previous reports of using GNR-based SERS as a tool to directly detect the expression of inflammatory molecules on inflamed macrophages. We also compared the detection sensitivity of our technique with other conventional methods, including fluorescent staining and ELISA as shown in Fig. 1.

## Experimental Sections

### Preparation of GNR Probe-Antibody Conjugates (GNR/4MBA@Anti-ICAM-1)

GNRs (1000 µL) obtained from Nanopartz were mixed with 10 mM 4-Mercaptobenzoic acid (4MBA, 10 µL) under vigorous stirring for 2 h and then centrifuged for 10 min at 6000×*g*. The pellet was re-dispersed in 300 µL milli-Q water, followed by the addition of 10 mg mL^−1^ poly(allylamine hydrochloride) (PAH;100 µL) and keeping gently stirred for 1 h. Next, the mixture was centrifuged for 10 min at 6000×*g*, and the pellet was re-dispersed in 500 µL of 5 % glutaraldehyde. Then, 25 µL of 0.1 mg mL^−1^ rat anti-mouse ICAM-1 antibody (monoclonal antibody; Biolegend) was added and incubated for 1 h on a stirrer (the antibody at this concentration is the optimal amount of ICAM-1 antibody that can stabilize particles). The mixture was again centrifuged, and the pellet was re-dispersed in 3 % bovine serum albumin (BSA) and incubated for 1 h. Finally, the conjugation was centrifuged and re-dispersed in borate buffer saline before being applied to cells. For the remainder of this paper, this conjugation will be referred to as GNR/4MBA@Anti-ICAM-1. 4MBA, BSA, glutaraldehyde, and PAH used in this study were purchased from Sigma-Aldrich.

### Cell Culture

The murine macrophage cell line (Raw264.7) was cultured in RPMI supplemented with 10 % fetal bovine serum (FBS, Nacalai Tesque) plus 1 % penicillin/streptomycin, Nacalai Tesque). Prior to use, cells were incubated at 37 °C for 24 h in an incubator with 5 % CO_2_.

### Cell Lysate Preparation

Cells were grown in a 24-well plate at a concentration of 1 × 10^5^ cells per well for 24 h. Next, cells were treated with lipopolysaccharide from *Escherichia coli* 055:B5 (LPS, 1 µg mL^−1^) for 1, 3, or 5 h. Following this, cells were washed twice with cold phosphate buffer saline (PBS) and were detached using cell scrapers. 500 µL of PBS was added to the cells, and then the cells were centrifuged at 15,800×*g* for 10 min. The cell pellet was lysed in NP40 cell lysis buffer (Invitrogen) containing 1 mM serine protease inhibitor (PMSF, Sigma-Aldrich) and protease inhibitor cocktail (Sigma-Aldrich; 500 µL per 5 mL of cell lysis buffer) and incubated at 4 °C for 30 min. After incubation, the sample was centrifuged at 15,800×*g* for 10 min. The supernatant was collected for measurement of ICAM-1 molecules using ELISA.

### Measurement of ICAM-1 Using ELISA

The measurement of ICAM-1 in the prepared lysates (Sect. 2.3) was performed using a commercial ELISA kit from Thermo Scientific, according to the manufacturer’s instructions.

### Cell Preparation for Fluorescent Staining

Raw264.7 cells were plated on a glass dish (Iwaki) at a concentration of 1 × 10^5^ cells per dish and grown for 24 h. Cells were then treated with 1 µg mL^−1^ of LPS for 1, 3, or 5 h. After LPS treatment, the cells were washed with PBS and fixed with 1.5 % paraformaldehyde for 15 min. The cells were washed again after fixing and incubated with 1 % BSA for 30 min. Then, cells were washed and incubated with 100 µL of fluorescein isothiocyanate (FITC)-conjugated anti-ICAM-1 antibody (Biolegend, 2.5 µg mL^−1^) in phosphate buffer saline with Tween20 (PBST buffer) and washed with PBST again after incubation. Finally, cells were washed with PBS before observation under a fluorescent microscope (FluoView, Olympus).

### SERS Measurement

Raw264.7 cells treated with LPS were prepared as described above. Next, cells were fixed with cold methanol for 10 min at approximately −20 °C. It is worth noting here that based on our experiences at our SERS measuring conditions, methanol fixation was used for SERS measurement because it helped reduce fluorescence background and provided better signal than when using paraformaldehyde. After fixing, cells were blocked with 3 % BSA for 1 h and then incubated with GNR/4MBA@Anti-ICAM-1 for 30 min at 37 °C (50 µL). Cells were washed with PBS before a SERS measurement was performed by Raman microspectroscopy (Nanophoton) using a 785-nm diode laser (power ~2.0 mW µm^−2^) as the excitation source. The sample was focused, and the SERS spectra were collected by a ×60 near infrared (NIR) microscope objective lens (1.0 NA, 2.8-mm working distance). An integration time of 3 s per line for all scans was used to collect SERS spectra. The measurement area was 134.8 µm in length and 28.5 µm in width. The SERS measurements of GNR/4MBA@Anti-ICAM-1 solution and Raman spectra of 4MBA powder were performed by means of the same laser excitation at a power of 30 mW with the integration time of 5 s. The spectra of SERS probe, measured for different cell conditions, were averaged from 3 to 4 different areas of each set of cells prepared on the glass dish. In general, five sets of cells for each treatment were used in an experiment. Therefore, all SERS spectra of each treatment were collected from 15 to 20 measurement areas. Human cervical cancer cells (HeLa cells) with and without treatment were used as negative control samples (nonspecific cells). The measurement area of HeLa cells was 134.3 µm in length and 57.6 µm in width. SERS spectral data were processed and extracted from SERS measurements by means of automated spectral detection and classification method as previously described by Pavillon et al. [28]. A low-value spatial position can be discarded through thresholding. In this work, SERS spectra were detected with a threshold value of 0.2. A baseline correction was also performed. All selected spectra were grouped in clusters based on spectral similarity using Pearson correlation with correlation coefficients above 0.7. The details of detecting tool software developed by Pavillon et al. [28] are already published in refs. [28, 29] and will not be discussed here.

### Transmission Electron Microscopy (TEM) Sample Preparation

The GNR probe-antibody conjugates (GNR/4MBA@Anti-ICAM-1) were deposited onto a Cu grid containing an elastic carbon substrate with a carbon thickness of ~20–30 nm (Okenshoji). The excess sample on the grid was removed by means of a sharp corner of filter paper. The sample was dried before being subjected to imaging with TEM (JEOL).

### Statistics

All statistical analysis was conducted by means of one-way ANOVA and Tukey–Kramer`s test at *P* < 0.05 using PHStat2 software (Pearson Eduacation).

## Results and Discussion

4MBA has been extensively used as a SERS reporter for generating a SERS signal [22, 30]. In general, the structure of 4MBA is composed of thiol and carboxyl groups. The attachment of 4MBA molecules on the surface of GNRs occurs through the thiol group of 4MBA. Following this reaction, the surfaces of our GNRs were coated with PAH to prevent the aggregation of GNRs, and then these GNRs were conjugated with antibodies. After preparation, the GNR probe-antibody conjugates (GNR/4MBA@Anti-ICAM-1) were characterized using TEM and Raman spectroscopy.

**Fig. 2:**
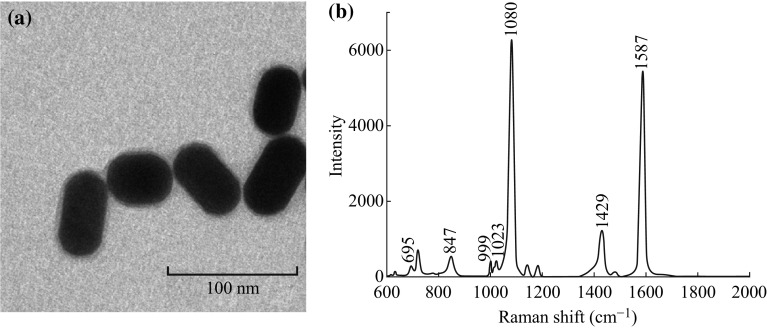
**a** TEM image of GNR/4MBA@anti-ICAM-1. **b** The average SERS spectrum of GNR/4MBA@anti-ICAM-1) measured using a 785-nm laser excitation with a power of 30 mW and 5-s integration time

**Fig. 3:**
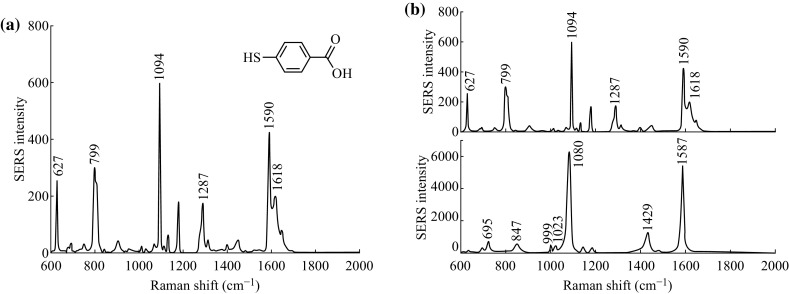
**a** The average Raman spectra of 4MBA powder. **b** The comparison of the SERS spectra of 4MBA powder (*top*) and GNR/4MBA@anti-ICAM-1 (*bottom*). All spectra were measured by a 785-nm laser excitation with a power of 30 mW and 5-s integration time

As seen in the TEM image (Fig. 2a), the size of GNR/4MBA@Anti-ICAM-1 particles is ~36 ± 1.37 nm in width and ~69 ± 3.12 nm in length. The distribution of GNR/4MBA@Anti-ICAM-1 particles at different aspect ratios is shown in Fig. S1. Using the conditions described in the Experimental Sect. 2.6, we were able to detect good SERS spectra for the GNR/4MBA@Anti-ICAM-1 probe particles (Fig. 2b). In Fig. 2b, there are observed intensely dominant peaks of 4MBA that can be attributed to the ring-breathing modes (at ~1080 and 1587 cm^−1^). The prominent observed bands in the spectrum are 695, 999, and 1023 cm^−1^. Compared with the Raman spectrum of 4MBA powder alone (Fig. 3), three new bands (695, 999, and 1023 cm^−1^) were detected in GNR/4MBA@Anti-ICAM-1 (Fig. 3a, b). It was previously reported that these new bands appear because the 4MBA molecules have been adsorbed on the surface of gold and that these new bands can be assigned as mono-substituted benzene derivatives [31]. The other two peaks that can be seen clearly are the peak at 1429 cm^−1^ from the *V*_s_(COO^−^) stretching mode [30] and the peak at ~847 cm^−1^, which can be assigned as the COO^−^ bending mode [22, 31].

**Fig. 4:**
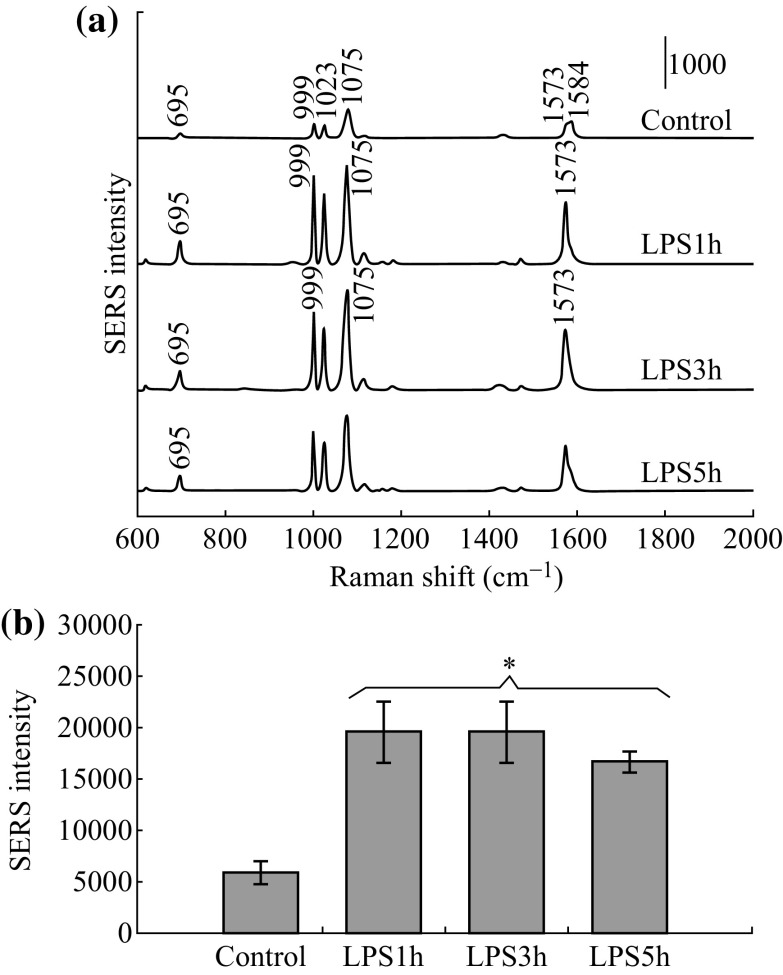
SERS spectra detected from Raw264.7 cells treated with LPS for different lengths of time (1, 3, and 5 h). Untreated cells were used as a control and GNR/4MBA@Anti-ICAM-1 particles were used as a target probe. **a** The SERS spectra were averaged from ~80 to 235 spectra detected for each condition. **b** The SERS intensities of the peak at ~1075 cm^**−**1^ detected in different treated and untreated cells. Comparisons between groups with a control sample were conducted by Tukey–Kramer`s test with significant set at *P* < 0.05

After collecting the spectra obtained using our prepared GNR/4MBA@Anit-ICAM-1 probe, we then applied this probe to detect the ICAM-1 on the cell surface of Raw264.7 cells that were left untreated or stimulated with LPS for varying lengths of time (1, 3, or 5 h). As seen in Fig. 4a, it is clear that the spectrum of SERS probe, which is specific to ICAM-1 on the surface of Raw264.7 cells stimulated with LPS, is significantly enhanced compared with the spectrum for the control cells (unstimulated Raw264.7 cells). The numbers of collected spectra of each treatment were in the range of ~80–230. Because the peak at ~1075 cm^**−**1^ shows the highest intensity, this peak was used to compare the SERS intensities of Raw264.7 cells treated with LPS at different incubation times and those of untreated cells (Fig. 4b). The SERS signal detected in cells treated with LPS for 1 h was significantly enhanced, ~3.3-fold higher than untreated cells. A published work using SERS probe particles with 4MBA as a reporter also found that the SERS signals detected in targeted cells were higher than that in non-targeted cells by ~3–4 folds [22, 31, 32].

**Fig. 5:**
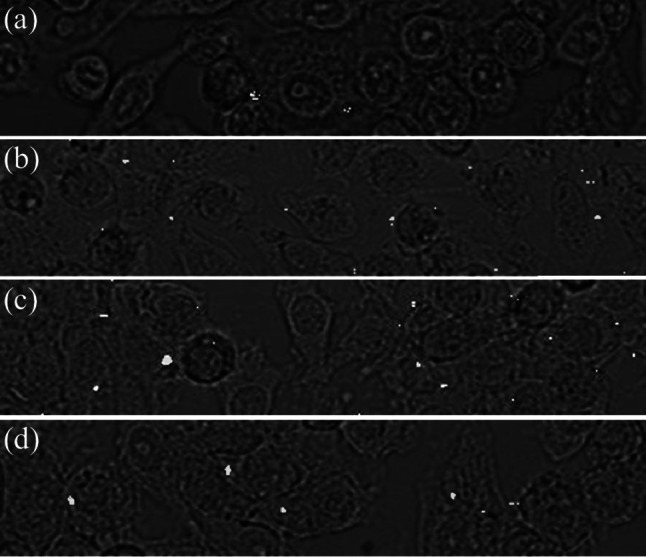
**a** The distribution of SERS signals (*yellow dots*) detected in untreated Raw264.7 cells and cells treated with LPS at different periods. Raw264.7 cells treated with LPS for 1 h (**b**), 3 h (**c**), and 5 h (**d**). The presence of *big yellow dots* observed in **c** and **d** implies the occurrence of high aggregation degree of particles. (Color figure online)

While the SERS probe technique has been used to distinguish between target and non-target cells, some work has also reported that the SERS probe technique can be used as a sensitive assay to detect targeted molecules at low concentrations [33, 34]. These reports, however, were not performed in cells. Here, we investigated the sensitivity of our GNR SERS probe at differentiating the target molecules (ICAM-1) expressed on the surface after treating cells with LPS for different lengths of time. As mentioned previously, our results show that the intensity of the peak at ~1075 cm^−1^ detected in cells treated with LPS were significantly increased compared with untreated cells (Fig. 4b). In addition to the differences in intensity observed for treated and untreated cells, a low distribution of SERS signals (yellow dots) in untreated cells was also observed (Fig. 5a). However, the intensity of the peak at ~1075 cm^**−**1^ in cells treated with LPS for 1 h was not different from that in cells treated with LPS for 3 h. A slight reduction of intensity in the peak at ~1075 cm^**−**1^ seems to occur in cells treated with LPS for 5 h (Fig. 4b).

**Fig. 6:**
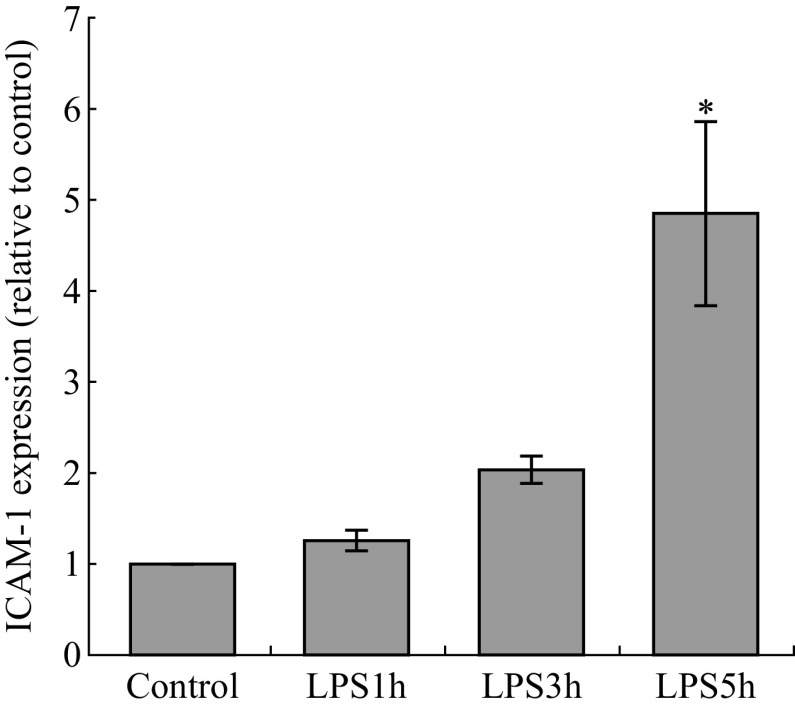
Relative ICAM-1 expression, as measured by ELISA, in Raw264.7 cells after treatment with LPS (1 µg mL^−1^) for 1, 3, and 5 h or left untreated (*control*). The *asterisk* represents significant ICAM-expression compared with a control sample. Comparisons between group were conducted by Tukey–Kramer`s test with significant set at *P* < 0.05

The SERS distributions detected in Raw264.7 cells under different conditions shown in Fig. 5 imply that an increased aggregation of particles might occur due to a high concentration of ICAM-1 antigen on the cell surface after stimulation with LPS. It has been reported that too much aggregation can decrease the intensity of SERS signal [35]. The expression of ICAM-1 in Raw264.7 cells, as measured by ELISA, confirms that the highest concentration of ICAM-1 is detected after treating cells with LPS for 5 h (Fig. 6). Therefore, the reduction of intensity of the peak at ~1075 cm^−1^ in cells treated with LPS might be due to a high aggregation of particles.

In general, Raw264.7 cells express ICAM-1 on their surfaces at a low concentration, but the expression of ICAM-1 can be increased by LPS stimulation. Figure 4a shows a small peak at ~1573 cm^**−**1^ in the SERS measurement of untreated Raw264.7 cells. This peak might occur as a result of the binding between GNR/4MBA@Anti-ICAM-1 particles and ICAM-1 molecules on the cell surface. Interestingly, the peak at ~1573 cm^**−**1^ was strongly enhanced after treating cells with LPS for different lengths of time (Fig. 4a). This indicates that the high number of ICAM-1 molecules on the cell surface might affect the binding of probe particles, resulting in enhanced peak intensity. Enhancement of the peak at ~1575 cm^**−**1^ was also reported after incubating HeLa cells with a polyvinylypyrrolidone (PVP)-coated probe that had 4MBA as a Raman reporter [36]. We also measured SERS signal in non-specific cells (HeLa and Vero cells). In this case, HeLa cells treated with LPS for 3 and 5 h were performed. For the Vero cells, cells were treated with LPS for 1 and 5 h. Non treated cells were also prepared for SERS measurement. As expected, SERS signals could not be detected in HeLa cells (Fig. S2). A few SERS signals were detected in all the treated and non-treated Vero cells (Fig. S3). This might occur from a small level of non-specific binding. As well, the examples of some single spectra from the map before averaging intensity values are provided in Fig. S4.

We compared our SERS probe technique for the detection of ICAM-1 expression with data collected using an ELISA. This revealed that an ELISA can detect a significant induction over untreated cells in the expression of ICAM-1 on Raw264.7 cells after they have been treated with LPS for 5 h (Fig. 6.) In contrast, the use of the GNR SERS probe particles prepared here shows that the expression of ICAM-1 molecules was significantly enhanced in cells treated with LPS over levels in control cells after only 1 h of treatment.

**Fig. 7:**
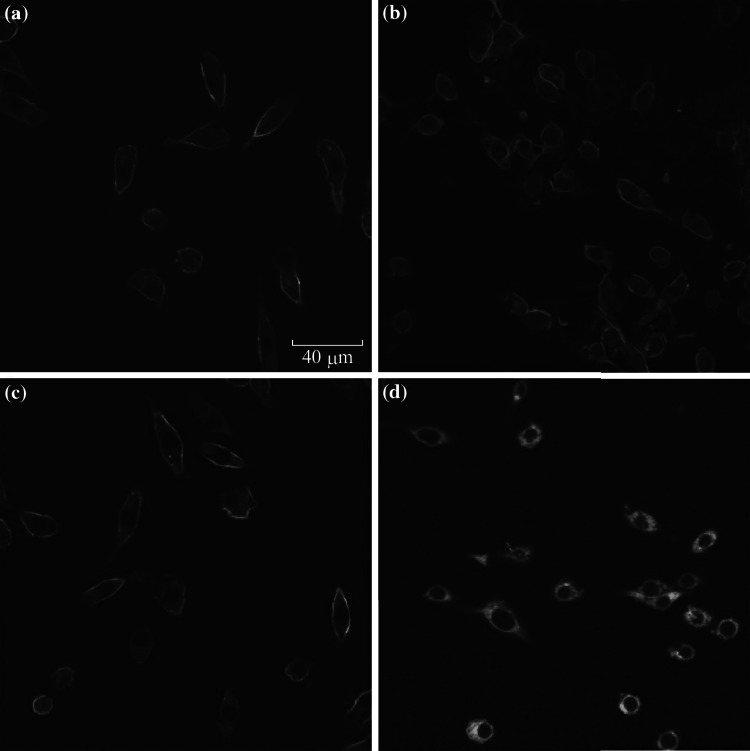
**a** Fluorescent images of untreated Raw264.7 cells (control) or cells treated with LPS (1 µg mL^−1^) for 1 h (**b**), 3 h (**c**), and 5 h (**d**). Cells were fixed and stained with FITC-ICAM-1 before the expression of ICAM-1 was observed using a fluorescent microscope

Lastly, we performed an additional test to show that the SERS probe GNRs method described here is more sensitive than the other conventional methods used for detecting changes of biomolecules in cells. In this case, a FITC-ICAM-1 antibody was used as an indicator to detect the expression of ICAM-1 on the surface of Raw264.7 cells. The signals of FITC attached to the ICAM-1 antibody could be clearly detected in cells treated with LPS for 5 h but not in cells treated with LPS for 1 or 3 h (Fig. 7). These results also show that the sensitivity of using fluorescent staining to detect the expression of ICAM-1 is much lower than the sensitivity provided by means of GNR/4MBA@Anti-ICAM-1 particles with Raman spectroscopy. While the ELISA and fluorescent dye-staining techniques could detect the signal of inflamed cells at a late time (5-h treatment), the relevant question is: why does the SERS signal of ICAM-1 decrease after treating cells with LPS for 5 h. The answer to this question is that it is due to more expression of ICAM-1 molecules on cell surfaces. This overexpression of ICAM-1 molecules in cells treated with LPS for 5 h (shown in Figs. 6, 7) caused more binding between GNR/4MBA@Anti-ICAM-1 and ICAM-1 molecules on cell surfaces leading to high aggregation and finally resulting in the decreasing of the SERS signal. It was reported previously by Ji et al. [36] that the interaction between the SERS probe and other molecules can lead to too much aggregation that causes the decrease of SERS signal. Furthermore, the saturation of the SERS signal might lead to a decrease in SERS signals.

## Conclusions

The combination of GNRs with a Raman reporter (4MBA) and specific antibody (ICAM-1) in the form of GNR/4MBA@Anti-ICAM-1 provides a strong SERS signal. This was used to detect the significant expression of ICAM-1 in cells stimulated with LPS for 1 h. In contrast, ELISA or fluorescent-labeling techniques could only detect the significant expression of ICAM-1 when cells were stimulated with LPS for 5 h. The greater sensitivity of the present GNR probes could provide a means to detect small changes early in the course of a disease. This would help to diagnose diseases earlier and potentially improve the treatment and management possibilities. Therefore, it may be worthwhile to develop the current GNR probe further in future. In particular, it would be useful to increase its sensitivity so that quantitative and time-resolved analyses of the biological molecules expressed in cells activated with stimulants could be obtained.

## Electronic supplementary material

Below is the link to the electronic supplementary material.

Supplementary material 1 (PDF 304 kb)
