# Supplementary material for: Detection of Adhesion Molecules on Inflamed Macrophages at Early-Stage Using SERS Probe Gold Nanorods
Source: Nanomicro Lett. 2016 Sep 23;9(1):8. doi: 10.1007/s40820-016-0111-7 (PMC6223776; doi:10.1007/s40820-016-0111-7)
Supplement: Supplementary file 1 — Supplementary material 1 (PDF 304 kb) [file 40820_2016_111_MOESM1_ESM.pdf]

Supporting Information for

Detection of Adhesion Molecules on Inflamed Macrophages at Early-Stage Using SERS Probe Gold Nanorods

Dakrong Pissuwan[1, 2,] * Yusuke Hattori[3]

[1]World Premier International Immunology Frontier Research Center, Osaka University, Osaka 5650871, Japan

[2]Materials Science and Engineering Program, Multidisciplinary, Faculty of Science, Mahidol University, Bangkok, 10400, Thailand

[3]Research Institute of Pharmaceutical Sciences, Musashino University, Tokyo 2028585, Japan

*****Corresponding author. E-mail: dakrong.pis@mahidol.ac.th

1 The Distribution of GNR/4MBA@Anti-ICAM-1 Particles

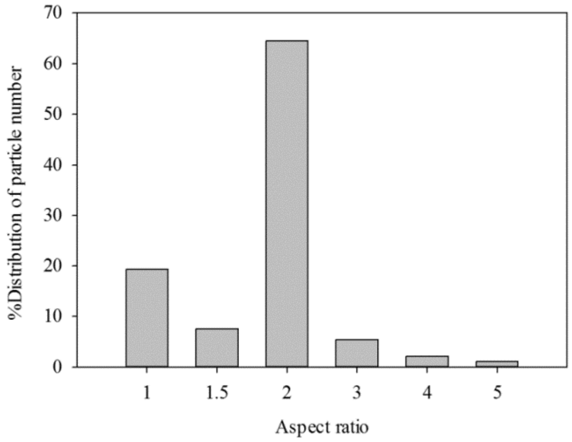

Fig. S1 The aspect ratio distribution of GNR/4MBA@Anti-ICAM-1 particles

2 SERS Measurement in HeLa Cells

HeLa cells treated with LPS for 3 and 5 h. Non-treated HeLa cells were also prepared as a control cell. No distribution of SERS signals was detected because of non-specific target cells (Fig. S2).

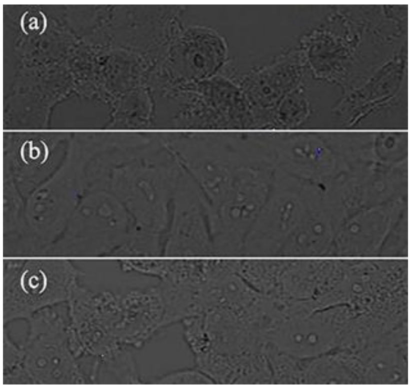

Fig. S2 HeLa cell images after SERS measurement under Raman spectroscopy; HeLa cells without treatment with LPS (**a**), HeLa cells treated with LPS for 3 h (**b**) and HeLa cells treated with LPS for 5 h (**c**). Cells were prepared and SERS measurement was performed following the experimental section No. 2.6 in the manuscript

3 SERS Measurement in Vero Cells

Vero cells treated with LPS for 1 and 5 h and non-treated Vero cells were used as a control cell. Similar numbers of yellow spots were detected in Vero cells treated with LPS (for 1 and 5 h) and in non-treated Vero cells. These small yellow spots might occur from non-specific binding of particles. However, there is no difference of SERS distribution signals. This should be due to without the specific target (ICAM-1) molecules on the cell surface to bind with GNR/4MBA@Anti-ICAM-1 particles (Fig. S2).

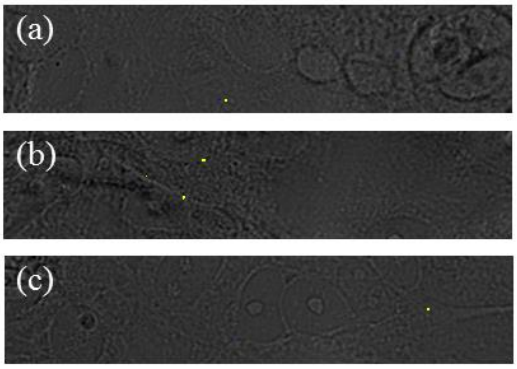

Fig. S3 Vero cell images after SERS measurement under Raman spectroscopy. Vero cells without treatment with LPS (**a**), Vero cells treated with LPS for 1 h (**b**) and 5 h (**c**). Cells were prepared and SERS measurement was performed following the experimental section No. 2.6 in the manuscript

4 Examples of Single Spectra of RAW 264.7 Cells after SERS Measurement

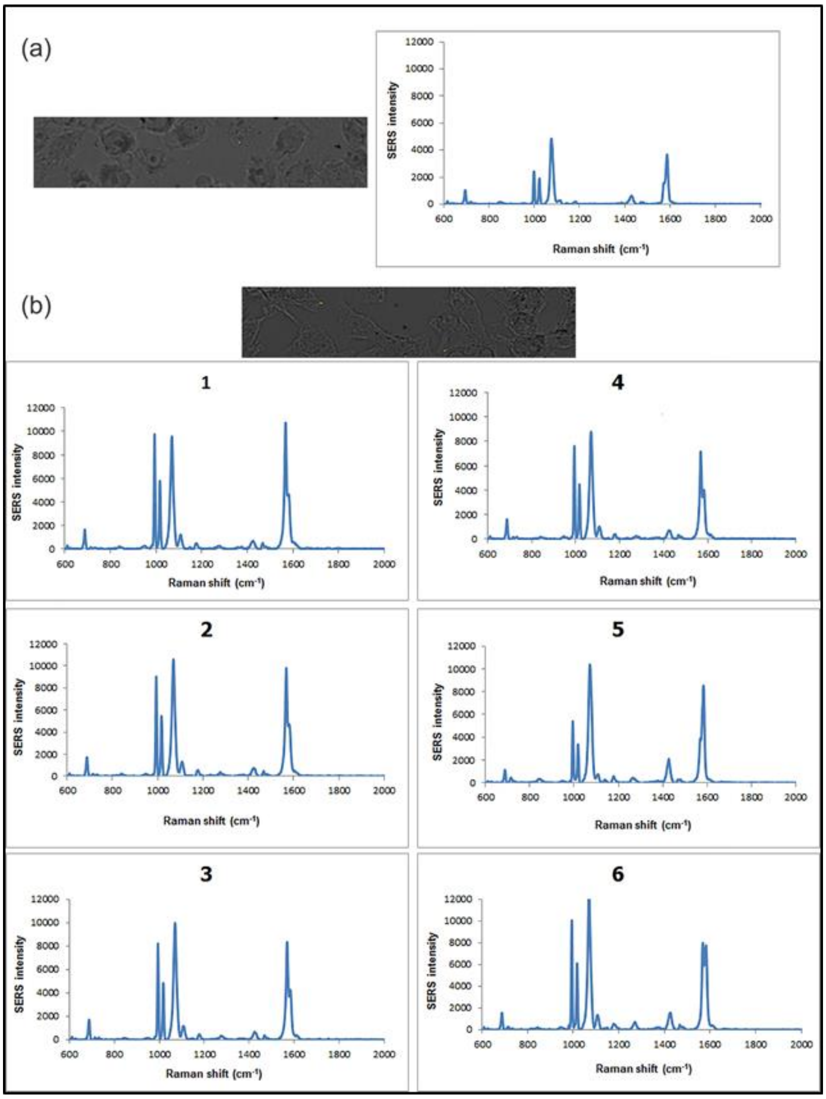

Fig. S4 Example of single spectra of RAW264. 7 cells after SERS measurement. The distribution of SERS signal was detected in RAW 264.7 cells without LPS treatment (**a**) and RAW264.7 cells treated with LPS for 1 h (**b**). The number of single collected spectra of RAW264.7 cells treated with LPS for 1 h was 6. Each single spectrum of RAW264.7 cells treated with LPS provides a strong SERS signal than that of without LPS treatment
